# Bringing prevost's sign into the third dimension: Artificial intelligence estimation of conjugate gaze adjusted length (CGAL) and correlation with acute ischemic stroke

**DOI:** 10.1097/MD.0000000000023330

**Published:** 2020-12-04

**Authors:** Hillel S. Maresky, Joseph M. Rootman, Miriam M. Klar, Max Levitt, Alexander P. Kossar, David Zucker, Michael Glazier, Shani Kalmanovich-Avnery, Richard Aviv, Birgit Ertl-Wagner, Sigal Tal

**Affiliations:** a Department of Radiology, Shamir Medical Center, Zerifin; b Sackler Faculty of Medicine, Tel Aviv University, Ramat Aviv, Israel; c Department of Radiology, Lewis Katz School of Medicine, Temple University, Philadelphia, Pennyslvania; d Department of Psychology, University of British Columbia, Kelowna, BC, Canada; e Department of Medicine, SUNY Downstate Health Sciences University, Brooklyn, New York; f Division of Urology, Department of Surgery, University of Ottawa, Ottawa, Ontario, Canada; g Department of Surgery, NewYork-Presbyterian Hospital/Columbia University Irving Medical Center, New York, New York; h Department of Interventional Neuroradiology, Shamir Medical Center, Zerifin, Israel; i Department of Radiology, The Ottawa Hospital and University of Ottawa, Ottawa; j Division of Neuroradiology, The Hospital for Sick Children, Toronto, Ontario, Canada.

**Keywords:** acute ischemic stroke, artificial intelligence, conjugate eye deviation, conjugate gaze adjusted length (CGAL), prevost sign

## Abstract

Conjugate gaze deviation is associated with acute ischemic stroke (AIS), although previously only measured on a 2D plane. The current study evaluates 3D imaging efficacy to assess conjugate gaze deviation and correlate direction and strength of deviation to neuro-clinical findings.

A retrospective analysis of 519 patients who had CT scans for suspected AIS at our institution. Direction and angle of eye deviation were calculated based on 2D axial images. Volumetric reconstruction of CT scans allowed for calculation of 3D conjugate gaze adjusted length (CGAL). Angle, direction, and vector strength of both 2D and 3D scans were calculated by an artificial intelligence algorithm and tested for agreement with hemispheric ischemia location. CGAL measurements were correlated to NIHSS scores. Follow up MRI data was used to evaluate the sensitivity and specificity of CGAL in the identification of AIS.

The final analysis included 122 patients. A strong agreement was found between 3D gaze direction and hemispheric ischemia location. CGAL measurements were highly correlated with NIHSS score (*r* = .72, *P* = .01). A CGAL >0.25, >0.28, and >0.35 exhibited a sensitivity of 91%, 86%, and 82% and specificity of 66%, 89%, and 89%, respectively, in AIS identification. A CGAL >0.28 has the best sensitivity-specificity balance in the identification of AIS. A CGAL >0.25 has the highest sensitivity.

Given CED's correlation with NIHSS score a 1/4 deviation in the ipsilateral direction is a sensitive ancillary radiographic sign to assist radiologists in making a correct diagnosis even when not presented with full clinical data.

## 1 Introduction

Conjugative shift of horizontal gaze towards the affected hemisphere, first described by Jean Louis Prevost in 1865^[1]^ and commonly referred to as conjugate eye deviation (CED), is a well-recognized occurrence in patients presenting with acute ischemic stroke (AIS) occurring in more than 55% of the computerized tomography (CT) scans taken upon hospital admission in AIS patients.^[1,2]^

Moreover, studies evaluating the clinical significance of CED in AIS patients have repeatedly demonstrated an association between the degree of deviation to stroke severity^[2–9]^ and poor prognostic outcomes.^[2–4,10–14]^ This is understandable, as CED-causing lesions reflect damage to cortical areas involved in the control of spatial attention, eye movements and the frontal eye fields,^[15,16]^ and thus presents as eye deviation ipsilateral to the affected brain hemisphere.^[17–20]^ It also explains the inclusion of eye deviation in clinical stroke severity diagnostic tools such as the NIH Stroke Scale (NIHSS).

There is no clear consensus as to the threshold for which CED becomes clinically diagnostic in AIS,^[7,8,14,20,21]^ although McKean and colleagues^[5]^ have documented CED >11.95° on CT imaging as having a 95.5% specificity but a sensitivity of only 17%. Furthermore, patients with CED >11.95° often have large areas of hypoattenuation^[2]^ and prior research has demonstrated that CED detection on CT scan does not increase reader identification of acute ischemic hypoattenuation if 4 or more Alberta Stroke Program early CT score (ASPECTS) zones are involved,^[21]^ thus limiting the added value of the CED >11.95° threshold. Similarly, NIHSS and CED degree are linked^[2–8]^ such that a patient with CED >11.95° usually presents with several other identifiable stroke symptoms, further decreasing CED contribution in AIS diagnosis. Lastly CED is not exclusively horizontal but can occur in any direction.^[19]^ Therefore, CED demonstrates potential as an adjunctive diagnostic tool in AIS diagnosis, the diagnostic utility of CED >11.95° is limited.

In our investigation to understand the discrepancy between this extensively documented concept and clinically useful results, we identified 2 variables that may require adjusting: previous studies evaluating CED used a 2D axis whereas the eye is a 3D structure and that the orbit size varies among individuals. We started investigating this in a pilot study, during which we observed that the combination of CED description from 3D CT images along with adjusting for globe radius, which we termed conjugate gaze adjusted length (CGAL), was highly sensitive and specific, and that a CGAL of >0.35 may be an appropriate threshold for assessing AIS.^[22]^ The current study continues this investigation and we devised a semi-automated artificial intelligence algorithm to shorten reconstruction time. We hypothesized that 3D CED assessment with adjustment for globe size will increase the efficacy of CED as a tool for the identification of AIS by decreasing the amount of deviation necessary to observe in patients with suspected AIS.

## 2 Ethics

This study was conducted with approval from our hospital's Institutional Review Board. Informed consent was waived for this study, as in accordance with the Declaration of Helsinki (IRB#: ASF-15-0056).

## 3 Methods

### 3.1 Case identification

This study retrospectively analyzed all patients undergoing emergent CT scans for suspected cerebrovascular accidents presenting to the emergency department between January 2012 and December 2013. Of these patients, those diagnosed with AIS were included in the study. Clinical diagnoses were made by the attending neurologists and radiologists. Exclusion criteria included patients diagnosed as a transient ischemic attack or with non-ischemic intracranial disease, CT scans that could not be 3D reconstructed due to excessive patient motion during the scan, patients who had previously undergone ocular lens surgery, patients who previously underwent enucleation of 1 or 2 eyes, patients under the age of 18, pregnancy, or patients withholding consent. Initial scans were performed within a mean time from hospital admission of 2 hours (SD = 1.8). When available, follow up CT and magnetic resonance imaging (MRI) scans, taken between 1 to 14 days after patient intake in select patients only when necessary to rule out post-stroke bleeding or stroke expansion were obtained for diagnosis comparison.

### 3.2 Imaging procedure

Initial and follow up CT scans were performed with identical, standard non-contrast CT protocol. CT scans were performed on a Philips Brilliance 64 MDCT machine (kVP 120, mAs 400 with dose modulation, FOV 225 mm axial slice thickness 3 mm, coronal and sagittal slice thickness 0.50 mm) and axial slices were obtained. Follow up MRI scans were performed using standard diffusion-weighted imaging (DWI) protocol on either Siemens Magnetom Aera 1.5T or Siemens Magnetom Skyra 3T MR imaging machines. The CT suite and procedure used in the present study had no features that would lead participants to direct patients gazes toward any particular direction.

### 3.3 Imaging post-processing, artificial intelligence & CGAL

**Figure 1:**
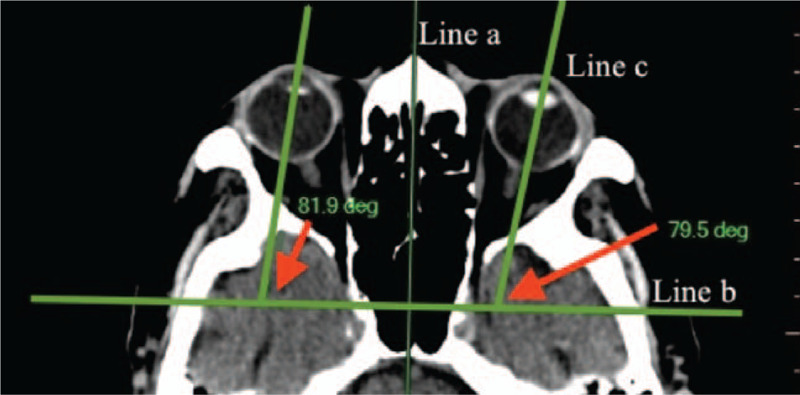
Measurement of CED. Line A is drawn through the midline. Line B, is drawn perpendicular to line A. Lines C are then drawn through the long axes of both lenses to create angles with line B from which the average CED may be calculated.

CT scans were assessed for 2D lateral deviation by a single reader. In accordance with the technique developed by Lesley et al,^[14]^ and utilized by McKean^[5]^ and others,^[20]^ 3 lines were drawn on the axial slice to facilitate measurement of the direction and angle of CED (Fig. 1): the first was drawn antero-posteriorly through the cranium at the midline (line a); the second line (line b) was drawn perpendicular to line a; finally a line was drawn through each lens at the long axis (line c) (Fig. 1a, b, c). Eye deviation for both the right (OD) and left (OS) eye was then measured by the angle found at the intersection of lines b and c. The presence of a deviation of >11.95° was used as the threshold for the identification of CED according to McKean et al.^[5]^

**Figure 2:**
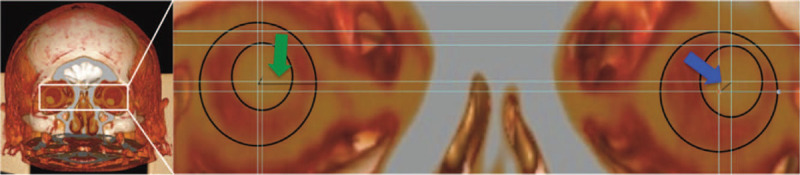
Measurement of CGAL. For each globe, vector length from the center of the lens to the center of the globe (blue arrow) is divided by globe radius (green arrow) to create CGAL measurements for both globe from which the average CGAL may be calculated.

CT scans were then reconstructed volumetrically using *Philips Intellispace Portal* and clipped coronally and tangentially to the frontal bone until the lens, roof of the orbit and zygomatic arch were exposed in the same plane. A high-resolution snapshot of the patients eyes was obtained at this clipping plane and stored as Tagged Image File Format (TIFF) (Fig. 2). This TIFF was then enhanced with *Adobe Photoshop* (contrast 100%, gain 20%) for image recognition software purposes. These enhanced snapshots were inputted into an artificial intelligence (AI) algorithm with *Amazon Rekognition* to calculate deviation direction, angle and vector length. Vector length was measured by the software from the center of the globe to the center of the lens (Fig. 2). The AI software recognized these landmarks by drawing gridlines on the superior, inferior, lateral, and medial borders of both spheres and then creating crosshairs at the centers of the gridlines. The vector length from the center of the lens to the center of the globe was then measured. The globe radius was calculated by the AI software by averaging 8 automatically-detected diameters and divided by 2. The vector length was then divided by globe radius by the AI algorithm and outputted as a size-adjusted measurement that we previously termed CGAL (See supplemental content for a video illustrating the 3D imaging process).

The vector angle was measured according to the *azimuth algorithm*, in which the object of interest (i.e., the globe) is placed on a 360° plane to allow for description of the vector angle. Angles between 0–180° were designated as positive, indicating a rightward directed gaze, and angles between 180°–360° were designated as negative, indicating a leftward directed gaze.

### 3.4 Statistical analysis

Statistical analyses were performed using SPSS (v.22). Kappa agreement analyses were conducted between right-right and left-left deviation and MCA territory on both 2D and 3D CT scans. A Pearson product moment correlation coefficient was derived to assess covariation between CGAL values and intake NIHSS scores. A *P* *<* .05 was considered significant. Finally, follow-up MRI data allowed for an identification of true positives and negatives. A ROC curve was constructed based on CGAL score and MRI follow up results, from which optimal CGAL values and their respective sensitivities and specificities were determined.

## 4 Results

A total of 519 patients underwent brain CT for suspected stroke in the given time period. Following removal of all patients who were negative for AIS and application of exclusion criteria, 122 patients were included in the final analysis. Follow up CT and MRI scans were available for 39 and 31 of these patients, respectively. Patient demographic characteristics are described in Table 1. The average time for image post-processing, image-recognition software and CGAL calculation was 3.6 minutes (SD = 0.8 minutes) per case.

**Table 1:** Study population characteristics.

Patient Characteristic	
Mean age	68.3 +/− 15.2
Male	74 (60.7%)
Mean Body Mass Index	32
Smoker	62 (50.8%)

**Figure 3:**
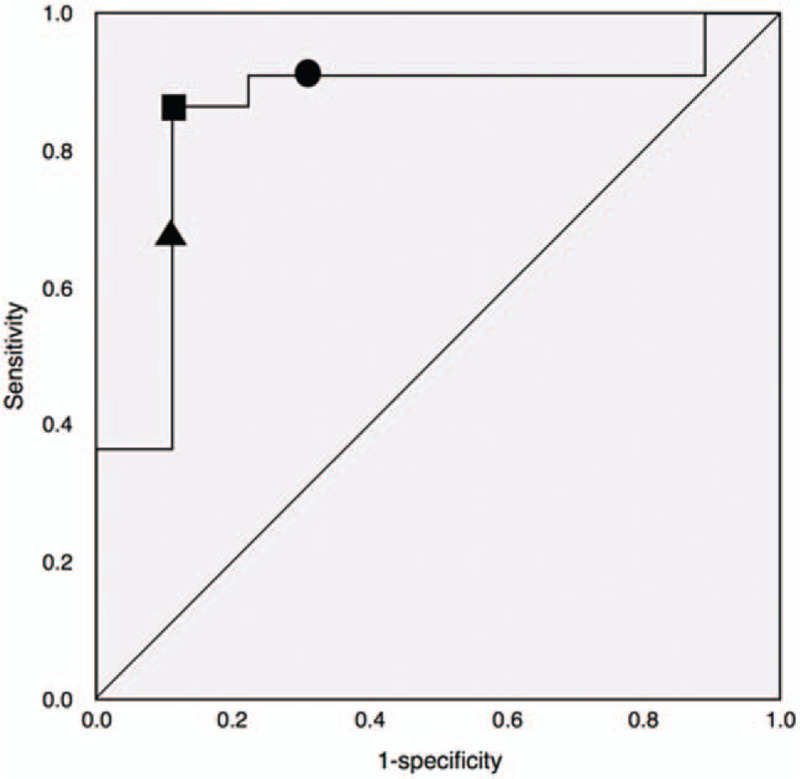
Empirical ROC curve illustrating the sensitivity and 1-specificity of gaze deviation in predicting AIS. Triangle = CGAL >0.35; square = CGAL >0.28; circle = CGAL >0.25.

Follow up MRI identified a high DWI signal in 22 of the 31 patients for which the data was available. Area under the curve (AUC) for CGAL prediction of AIS was = 0.85 (Fig. 3). Three CGAL thresholds were evaluated for sensitivity and specificity. These included CGAL >0.35 due to its significance in our pilot study,^[22]^ CGAL >0.28 because it displayed the best balance of sensitivity and specificity, and CGAL >0.25 because it displayed the highest sensitivity and is an easy value for radiologists to work with. The details of the sensitivity and specificity analysis are reported in Table 2.

**Table 2:** Sensitivity and specificity for CGAL values of interest.

CGAL Measurement	Sensitivity (True Positives)	Specificity (True Negatives)
>0.25	91% (20)	66% (6)
>0.28	86% (19)	89% (8)
>0.35	82% (18)	89% (8)

CGAL >0.28 was calculated in 92 patients (89%). CGAL measurements indicated that 45 patients (44%) ocular deviation was directed rightwards whereas 46 patients (45%) exhibited leftward deviation. These deviations demonstrated strong Kappa agreements with clinical MCA territory with rightwards deviation agreements found to be *k* = .85 and leftwards deviation agreements found to be *k* = .72. A 2D horizontal gaze >11.95° was observed in 78 patients (76%). 2D CED measurements demonstrated that 41 patients (40%) exhibited rightwards deviation and 37 patients (36%) leftwards with deviation-MCA territory agreements found to be *k* = .74 and *k* = .67 for rightwards and leftwards deviation respectively.

Horizontal gaze deviation was noted in 22 of the 39 CT follow up scans (56%). Additionally, a CGAL value greater than 0.28 was noted in 16 (42%) of these follow up CT scans. Kappa agreements for deviation direction-MCA ischemic territory were *k* = .39 for rightwards deviation and *k* = .45 for leftwards deviation. This decrease in CED during the subacute phase compared to initial CGAL measurements taken during the acute stroke phase suggests that the CED observed in the initial scans was a result of AIS and not due to random deviation.

**Figure 4:**
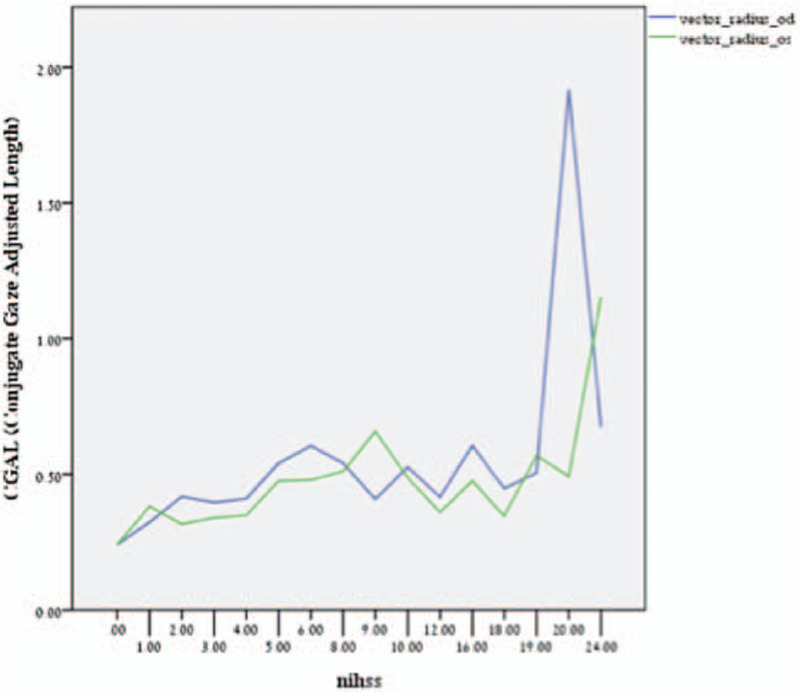
Pearson Product Moment Correlations between NIHSS scores and CGAL measurements for each globe. The blue line represents the right eye (OD) and the green line represents the left eye (OS). NIHSS scores and their associated stroke severity interpretation are as follows: 0 = No stroke symptoms, 1-4 = Minor stroke, 5-15 = Moderate stroke, 16–20 = Moderate to severe stroke, 21–42 = Severe stroke.

CGAL deviation values were found to be strongly and significantly correlated with NIHSS score (Fig. 4) for both the left (*r* = .59, *P* < .01) and right eye (*r* = .64, *P* < .01). Not surprisingly, when the individual eye CGAL scores were averaged the correlation remained significant (*r* *=* .72, *P* *=* .01).

## 5 Discussion

The present study is the first to demonstrate the efficacy, utility, and validity of 3D CT image assessments for the presence of CED in the evaluation of AIS. Importantly, a CGAL measurement >0.28 was selected for this study based on its strong balance of sensitivity (86%) and specificity (89%). Ischemic hemisphere and gaze direction showed strong agreement and, moreover CGAL severity and the NIHSS scale were highly correlated. These findings highlight the validity of CGAL measurements in identifying AIS and quantifying its severity.

The high sensitivity and specificity of a CGAL measurement >0.28 calculated in the current study reflect positively on the use of CGAL measurements in that the presence of both is a hallmark of a strong diagnostic test. Furthermore, the sensitivity rating, in particular, far exceeds that reported in McKean and colleagues evaluation of 2D CT CED efficacy^[5]^ as well as other standard radiographic signs of AIS such as the detection of hypodensity in greater than 33% of the MCA territory.^[23]^ This suggests that 3D CT image assessments for the presence of CED in suspected AIS patients is an effective diagnostic sign.

Importantly, the present study demonstrates that, in the context of initial neuroimaging scans, a CGAL measurement >0.28 is a highly specific and sensitive indirect sign of AIS. At this early point in stroke code protocol direct radiographic signs of AIS, such as parenchymal changes or ischemia, are occasionally undetectable. ^[24]^ In the case where an initial CT scan provides unsubstantial evidence for the diagnosis of AIS, an additional follow up CT scan is often performed which not only subjects the patient to unnecessary radiation, ^[25]^ but also consumes valuable time. Indirect CT AIS findings, such as CED, can guide clinical decision making in the absence of direct findings and lack of clinical data available to radiologists and allow for progress in patient treatment without the need for a secondary scan. The elimination of this secondary scan is extremely important as nearly 14 billion synapses and 1.9 million neurons are lost for every minute of brain ischemia.^[26]^ Considering the time wasted if imaging is reperformed, having a snapshot of the patients eyes in 3D, especially in the absence of a complete patient history, may aid a radiologist to actively search for signs of ipsilateral ischemia. When presented with strong 3D CGAL deviation (i.e., over 1/4) this may alert the radiologist in an otherwise equivocally normal non-contrast head CT to search further and to decide whether or not the scan is indeed completely normal. On the other hand, in the absence of dense vessel signs or obvious parenchymal hypoattenuation, if there is no significant CGAL, this could act as an additional layer of evidence for the radiologist to soundly interpret the scan as negative.

In applying the findings of this paper there are a few important considerations. First, non-contrast CT is known to have a margin of error of ∼1 mm, thereby limiting the spatial resolution when obtaining measurements <1 mm. Moreover, as noted, sensitivity is of greater value then specificity in the context of AIS, therefore in accounting for the limitations of CT spatial resolution it is reasonable to lean on more sensitive thresholds. Finally, a full 3D reconstruction and measurement may be time consuming. Thus, although ROC curve analysis indicated that a CGAL >0.28 exhibits the best balance of sensitivity and specificity, clinicians may save time and reduce the risk of false negatives by considering a rule of thumb wherein an approximate 1/4 deviation in the ipsilateral direction is considered a sensitive (91%) ancillary sign for AIS.

Beyond the excellent sensitivity and specificity of a CGAL measurement >0.28, the validity of 3D CT imaging for the assessment of CED in AIS patients is further strengthened by multiple findings in the present study. In concordance with prior investigations of CED in AIS patients, as well as the evaluation of 2D scans used in the present study, there was a high agreement between the direction of deviation noted via 3D CT evaluation and clinical MCA ischemic territory.^[17–20]^ Moreover, the present study identified a strong correlation between CGAL measurements and the NIHSS scale scores, which builds on another well established association between CED and stroke symptomology.^[2–9]^ The fact that the 3D CT assessment of CED is in line with prior 2D CT research and empirically validated stroke scales is a good indication that the 3D method has a similar level of validity to the classically used 2D method as a radiographic sign of AIS.

Interestingly, a CGAL >0.28 was found in near equal proportions within both left and right CED gaze direction patients. Although rightward directed gaze has typically thought to be far more common in AIS patients,^[4,10,11,19]^ both the present study and other recent literature^[7]^ casts doubt on this finding. Because deviation is typically ipsilateral to the damaged hemisphere,^[17–20]^ as it generally was in the present study, this finding suggests that CED is not specific to right hemispheric strokes.

The present study is not without limitations. Importantly, gaze deviation during neuroimaging can occur spontaneously and without clinical reason. This sort of random deviation has the potential of skewing the results in any CED study, and while our study is not impervious to this error, follow up CT scans obtained in the present study indicated far less severe deviation and worse deviation-MCA territory agreement than initial CT scans. This contrast between initial and follow up CT scans suggests that CED had subsided in our sample (as is typically the case with CED caused by AIS),^[11]^ and thus the initial CT measurements taken in the present study were likely not due to random patient eye deviation. Finally, it is worth noting that although the sensitivity of a CGAL measurement >0.28 is exceptionally high, the specificity of this tool is lower than was found in McKean and colleagues 2D CED validation study. Nonetheless, the value of a tool is typically thought to be best represented in the balance of sensitivity and specificity. Moreover in the context of AIS, where the prompt identification of the condition is critical to patient outcome, a tool that is good at identifying true positives is more useful than a tool that would miss these positives in favor of identifying true negatives. Future research could expand on the present study by evaluating patient long-term prognostic and MCA ischemia volume data in relation to CGAL severity. This information would help to further validate 3D CT assessments of CED in AIS patients and increase understanding of the overall utility of the tool.

## 6 Conclusion

In conclusion, a CGAL value >0.28 found in the 3D CT assessment of CED in patients with suspected AIS is a quick, accurate and useful addition to other radiographic signs. Such a tool can aid in the radiologists decision making processes in the context of otherwise negative or ambiguous CT scan imaging, especially given the lack of clinical data available to radiologists, and, therefore, speed up a protocol where time is of the absolute essence.

## Acknowledgments

We thank Dr. Paul Gottlieb for his encouragement and support of a research-driven atmosphere.

## Author contributions

**Conceptualization:** Hillel S Maresky, Sigal Tal.

**Data curation:** Hillel S Maresky, Sigal Tal.

**Formal analysis:** Hillel S Maresky, Joseph M. Rootman, Miriam M. Klar.

**Funding acquisition:** Hillel S Maresky.

**Investigation:** Hillel S Maresky.

**Methodology:** Hillel S Maresky, Max Levitt, Alexander P. Kossar, David Zucker, Sigal Tal.

**Project administration:** Hillel S Maresky, Miriam M. Klar.

**Resources:** Hillel S Maresky.

**Software:** Hillel S Maresky.

**Supervision:** Hillel S Maresky, Sigal Tal, Miriam M. Klar.

**Validation:** Hillel S Maresky.

**Writing – original draft:** Joseph M. Rootman.

**Writing – review & editing:** Hillel S Maresky, Joseph M. Rootman, Miriam M. Klar, Michael Glazier, Shani Kalmanovich-Avnery, Richard Aviv, Birgit Ertl-Wagner, Sigal Tal.

## Supplementary Material

Supplemental Digital Content

## Supplementary Material

Supplemental Digital Content
